# Relationship among nephron number, cortical volume, and glomerular pressure in healthy kidney donors

**DOI:** 10.14814/phy2.70462

**Published:** 2025-07-25

**Authors:** Hiroki Nobayashi, Go Kanzaki, Takaya Sasaki, Rina Oba, Yusuke Okabayashi, Kotaro Haruhara, Kentaro Koike, Akimitsu Kobayashi, Izumi Yamamoto, Nobuo Tsuboi, Takashi Yokoo

**Affiliations:** ^1^ Division of Nephrology and Hypertension, Department of Internal Medicine The Jikei University School of Medicine Tokyo Japan

**Keywords:** filtration, hemodynamics, kidney, nephron, tubule

## Abstract

Glomerular hypertension is a critical factor in kidney disease progression. Traditionally, nephron number (Nglom) was considered a determinant of glomerular hemodynamics, but emerging evidence suggests a more substantial impact of renal structural factors. This cross‐sectional study aimed to evaluate the relationship between renal histopathological parameters and intraglomerular hydrostatic pressure (Pglo) in healthy donors. Retrospective analyses were conducted on 60 healthy living donors who donated a kidney between January 1, 2007, and September 30, 2022. The non‐sclerotic glomerular number (Nglom_NSG_), glomerular volume, cortical volume, and other histopathological parameters were evaluated. Pglo was estimated using Gomez's equation. Pglo was positively associated with cortical volume (standardized regression coefficient [*β*] = 0.34, *p* = 0.04) but not with Nglom_NSG_ (*β* = 0.12, *p* = 0.44). Cortical volume was inversely associated with afferent arteriolar resistance (*β* = −0.33, *p* < 0.01). These findings suggest that an increase in cortical volume, independent of nephron number, may contribute to an increase in Pglo through a reduction in afferent arteriolar resistance. These results refine current models of glomerular hemodynamics, highlighting the importance of structural renal evaluation, including cortical volume, in assessing early chronic kidney disease risk.

## 1 INTRODUCTION

Chronic kidney disease (CKD) affects approximately 8%–16% of the global population and increases the risk of cardiovascular events and mortality (Chen et al., 2019; Di Angelantonio et al., 2010). Common risk factors include diabetes, hypertension, glomerulonephritis, infections, and environmental exposures (Jha et al., 2013). Despite its diverse etiologies, nephron loss is considered a fundamental mechanism driving CKD progression (Romagnani et al., 2017).

Brenner et al. proposed that a reduced nephron number, whether congenital or acquired, predisposes individuals to hypertension and glomerular hyperfiltration, eventually leading to glomerular injury and nephron depletion (Brenner et al., 1988). However, direct evaluation of nephron number and intraglomerular pressure (Pglo) in living individuals is technically challenging.

Recent advances have enabled the estimation of total glomerular number (Nglom_TOTAL_) in living kidney donors by combining computed tomography (CT) angiography and stereological analysis of renal biopsy specimens (Denic, Mathew, et al., 2017). This approach has identified height as a predictor of glomerular number. Similar techniques validated in Japanese living donors have shown consistency with the autopsy‐based physical fractionator/fractionator methods (Kanzaki et al., 2017). Further research has suggested that the single‐nephron glomerular filtration rate responds to variations in dietary protein intake in these donors (Oba et al., 2020).

Gomez originally developed equations to indirectly estimate glomerular hemodynamics, including Pglo (Gomez, 1951). More recent research has correlated Pglo, estimated using Gomez's equations, with metabolic parameters, such as serum uric acid and insulin resistance (Tsuda et al., 2018; Uedono et al., 2017). Nevertheless, the direct relationship between estimated hemodynamic parameters and renal morphology in living donors remains unclear.

This study aimed to investigate whether Pglo, as estimated using Gomez's formula, is related to nephron number, glomerular volume, cortical volume, and other morphological factors in a cohort of living kidney donors.

## 2 MATERIALS AND METHODS

### 2.1 Participants

**FIGURE 1:**
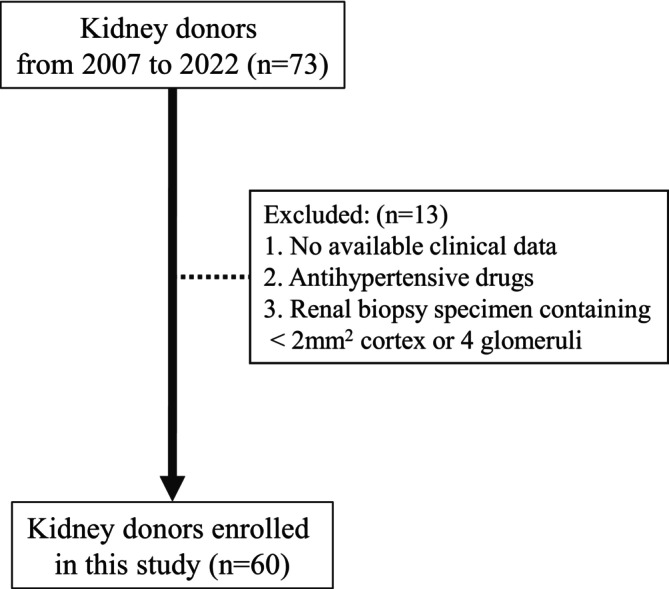
Patient selection. Donors who underwent kidney biopsy at the time of donation are selected. Those taking antihypertensive drugs, kidney biopsy specimens with <2 mm^2^ cortex and/or <4 non‐sclerotic glomeruli, and those with missing clinical data are excluded.

This retrospective cohort study included donors who underwent enhanced CT imaging and kidney biopsies at the time of donation at the Jikei University Hospital in Tokyo, Japan, between January 1, 2007, and September 30, 2022. All kidney donors were selected according to the Amsterdam Forum guidelines (Delmonico & Council of the Transplantation, 2005). All participants exhibited normal glucose tolerance. Hypertension was defined as systolic blood pressure ≥ 140 mmHg and/or diastolic blood pressure ≥ 90 mmHg, measured during a systemic medical checkup before kidney transplantation. Individuals with hypertension who were taking antihypertensive drugs were excluded due to their effect on intraglomerular hemodynamics. Kidney biopsy specimens with <2 mm^2^ cortex and/or <4 non‐sclerotic glomeruli were excluded, based on the findings of previous studies (Denic, Mathew, et al., 2017; Sasaki et al., 2019) (Figure 1). This study was approved by the ethics review board of the Jikei University School of Medicine (approval no. 30–060, 9081). Furthermore, the study followed the tenets of the Declaration of Helsinki. Because this was a retrospective cohort study, information on the research plan and the possibility of publication was proposed, and an opportunity to opt out was provided; therefore, individual informed consent was not required.

### 2.2 Measurements

General demographic data, including age, height, body weight, medical history, treatment, and blood pressure, as well as laboratory measurements, were extracted from medical records prior to donation. Urine samples were collected over a 24‐h period. The body mass index (BMI) was calculated by dividing the body weight by the height squared. The estimated glomerular filtration rate (eGFR) was derived from serum creatinine levels measured 1 day prior to transplantation using the Japanese Society of Nephrology Formula (Male: eGFR = 194 × Creatinine^−1.094^ × Age^−0.287^, Female: eGFR = 194 × Creatinine^−1.094^ × Age^−0.287^ × 0.739) with units of mL/min per 1.73 m^2^ (Matsuo et al., 2009). As standardization based on body surface area (BSA) is inappropriate, the glomerular filtration rate (GFR) was calculated using the formula: GFR = eGFR × BSA/1.73, with units of mL/min to evaluate individual hemodynamics. BSA was calculated using the Dubois formula as follows: BSA (m^2^) = body weight^0.425^ × height^0.725^ × 0.007184 (Du Bois & Du Bois, 1989). Effective renal plasma flow (ERPF) was calculated from 99mTc–MAG3 clearance values using a previously validated equation: effective renal plasma flow (mL/min) = 1.86 × 99mTc–MAG3 clearance + 4.6 (Muller‐Suur et al., 1991). Renal blood flow (RBF) can be calculated from the ERPF and hematocrit using the standard formula RBF = ERPF/(1–Ht). The total number of glomeruli per kidney (Nglom_TOTAL_) was estimated using a previously reported method (Denic, Mathew, et al., 2017). This involved multiplying the glomerular density of the kidney biopsy specimens collected at the time of kidney donation by the total renal cortical volume obtained from enhanced CT images. Cortical volumes were measured using ITK–SNAP software (version 1.1, University of Pennsylvania, Philadelphia, PA, USA) (Yushkevich et al., 2006). The total number of glomeruli was divided by two to determine the number per kidney, by 1.43 to account for the decrease in tissue volume due to paraffin embedding, and by 1.268 to account for the decrease in volume due to loss of tissue perfusion pressure. The degree of non‐sclerotic glomerular profile was calculated for each kidney specimen and multiplied by Nglom_TOTAL_ to obtain the number of non‐sclerotic glomeruli (Nglom_NSG_). Glomerular volume (Vglom) was calculated based on the mean area of the glomerular capillary area, and total glomerular volume per kidney was calculated by multiplying glomerular volume by Nglom_NSG_. The details of the calculation of glomerular number and morphology were described in our previous report (Sasaki et al., 2019).

The glomerular hydrostatic pressure (Pglo), afferent arteriolar resistance (Ra), and efferent arteriolar resistance (Re) were calculated using the Gómez formula (Gomez, 1951). According to the original publication, the Gomez formula is as follows:

∆PF=GFR/KFG

$$\text{Pglo}=\text{∆}\mathrm{PF}+P_{\mathrm{BOW}}+\mathrm{\pi G}$$

$$\mathrm{\pi G}=5\times \left(\mathrm{CM}–2\right)$$

$$\mathrm{CM}=\mathrm{TP}/\mathrm{FF}\times \ln\left(1/\left(1–\mathrm{FF}\right)\right)$$

$$\mathrm{Ra}=\left(\left(\mathrm{MBP}–\text{Pglo}\right)/\mathrm{RBF}\right)\times 1,328$$

Re=GFR/KFG×RBF–GFR×1,328

where ⊿PF is the filtration pressure across the glomerular capillary. KFG (the gross filtration coefficient) was estimated as 0.0406 mL/s·mmHg per kidney, P_BOW_ (the hydrostatic pressure in Bowman's space) was estimated as 10 mmHg, and πG (the oncotic pressure within the glomerular capillaries) was obtained from the plasma protein concentration within the glomerular capillaries (CM) and calculated from the total protein concentration (TP) and filtration fraction (FF). FF was calculated by dividing GFR by RPF. Ra and Re were calculated using Ohm's law and the conversion factor of dyne·s·cm^−5^.

### 2.3 Statistical analyses

The baseline characteristics of the overall cohort of 60 participants, as well as those categorized in the low, intermediate, and high Pglo groups, according to the Pglo tertiles were documented. Continuous variables are presented as medians with interquartile ranges, whereas categorical variables are expressed as frequencies with percentages. Continuous variables were compared among the three groups using the Kruskal–Wallis test. Nominal variables were assessed using the Fisher's exact test. Variables with significant results were further analyzed with Bonferroni‐adjusted pairwise comparisons, with *p* < 0.05 considered significant. Spearman's rank correlation coefficient was utilized to assess correlations between two variables. Multivariable linear regression analyses were conducted to evaluate the factors contributing to Pglo, Ra, and Re. Statistical significance was defined as *p* < 0.05. All statistical analyses were performed using SPSS v.29.0 (IBM Corp., Armonk, NY, USA).

## 3 RESULTS

### 3.1 Clinical characteristics

Table 1 summarizes the clinical characteristics of the 60 donors, including demographic, laboratory, and intraglomerular hemodynamic data. The median patient age was 57 years, and 32% of the participants were male. There were seven (12%) individuals with mild hypertension among the 60 donors, but none with diabetes. The median serum creatinine level and the eGFR were 0.66 mg/dL and 76 mL/min/1.73 m^2^, respectively. eGFR and Re in the high Pglo group were significantly higher than those in the low Pglo group, and eGFR in the intermediate Pglo group was also higher than that in the low Pglo group. The serum creatinine level in the high Pglo group was lower than that in the low and intermediate Pglo groups. No significant differences were observed among the three groups for the other clinical characteristics.

**TABLE 1:** Clinical characteristics of living kidney donors at the time of donation in the entire cohort, and in groups according to tertiles of Pglo.

	All *n* = 60	Low Pglo (46.6–51.4 mmHg) (*n* = 20)	Intermediate Pglo (51.5–55.8 mmHg) (*n* = 20)	High Pglo (55.8–78.4 mmHg) (*n* = 20)	*p* Value
Age, years	57 (49–63)	58 (51–68)	55 (48–61)	57 (48–65)	0.46
Male, *n* (%)	19 (32)	6 (30)	8 (40)	5 (25)	0.69
Height, m	1.61 (1.55–1.66)	1.59 (1.53–1.61)	1.61 (1.57–1.72)	1.61 (1.58–1.66)	0.15
Body weight, kg	59 (53–68)	55 (50–63)	63 (55–69)	61 (52–70)	0.09
BMI, kg/m^2^	23 (21–25)	23 (21–25)	24 (21–26))	23 (21–27)	0.87
SBP, mmHg	120 (108–132)	123 (112–132)	112 (103–126)	130 (108–137)	0.10
DBP, mmHg	70 (62–78)	69 (60–77)	70 (61–75)	73 (62–82)	0.36
Hypertension, *n* (%)	7 (12)	3 (15)	1 (5)	3 (15)	0.68
Creatinine, mg/dL	0.66 (0.56–0.80)	0.74 (0.63–0.91)	0.67 (0.60–0.84)	0.54 (0.49–0.68)^a^, ^b^	< 0.01
eGFR, mL/min/1.73m^2^	76 (67–90)	67 (61–73)	77 (69–84)^a^	93 (86–106)^a^, ^b^	< 0.01
ERPF, mL/min	372 (291–460)	298 (273–481)	384 (341–471)	359 (309–447)	0.53
Pglo, mmHg	53.7 (51.8–57.3)	50.8 (48.9–51.9)	53.7 (53.2–55.4)	59.1 (57.1–61.3)	
Ra, dyne·s·cm^−5^	4178 (2717–5679)	5593 (2570–6797)	3882 (2717–5144)	4071 (2703–4939)	0.07
Re, dyne·s·cm^−5^	2238 (1742–2744)	2197 (1344–2558)	2025 (1596–2547)	2663 (2235–3422)^a^, ^b^	< 0.01
HbA1c, %	5.5 (5.2–5.8)	5.6 (5.1–5.8)	5.5 (5.4–5.7)	5.7 (5.2–5.9)	0.82
UPE, mg/day	31 (14–49)	24 (13–41)	41 (21–55)	25 (9–50)	0.13

Abbreviations: BMI, body mass index; DBP, diastolic blood pressure; eGFR, estimated glomerular filtration rate; ERPF, estimated renal plasma flow; HbA1c, hemoglobin A1c; Pglo, intraglomerular hydrostatic pressure; Ra, afferent arteriolar resistance; Re, efferent arteriolar resistance; SBP, systolic blood pressure; UPE, urine protein excretion.

^a^ Bonferroni adjusted *p* < 0.05 versus low Pglo.

^b^ Bonferroni adjusted *p* < 0.05 versus intermediate Pglo.

### 3.2 Histopathological characteristics

**FIGURE 2:**
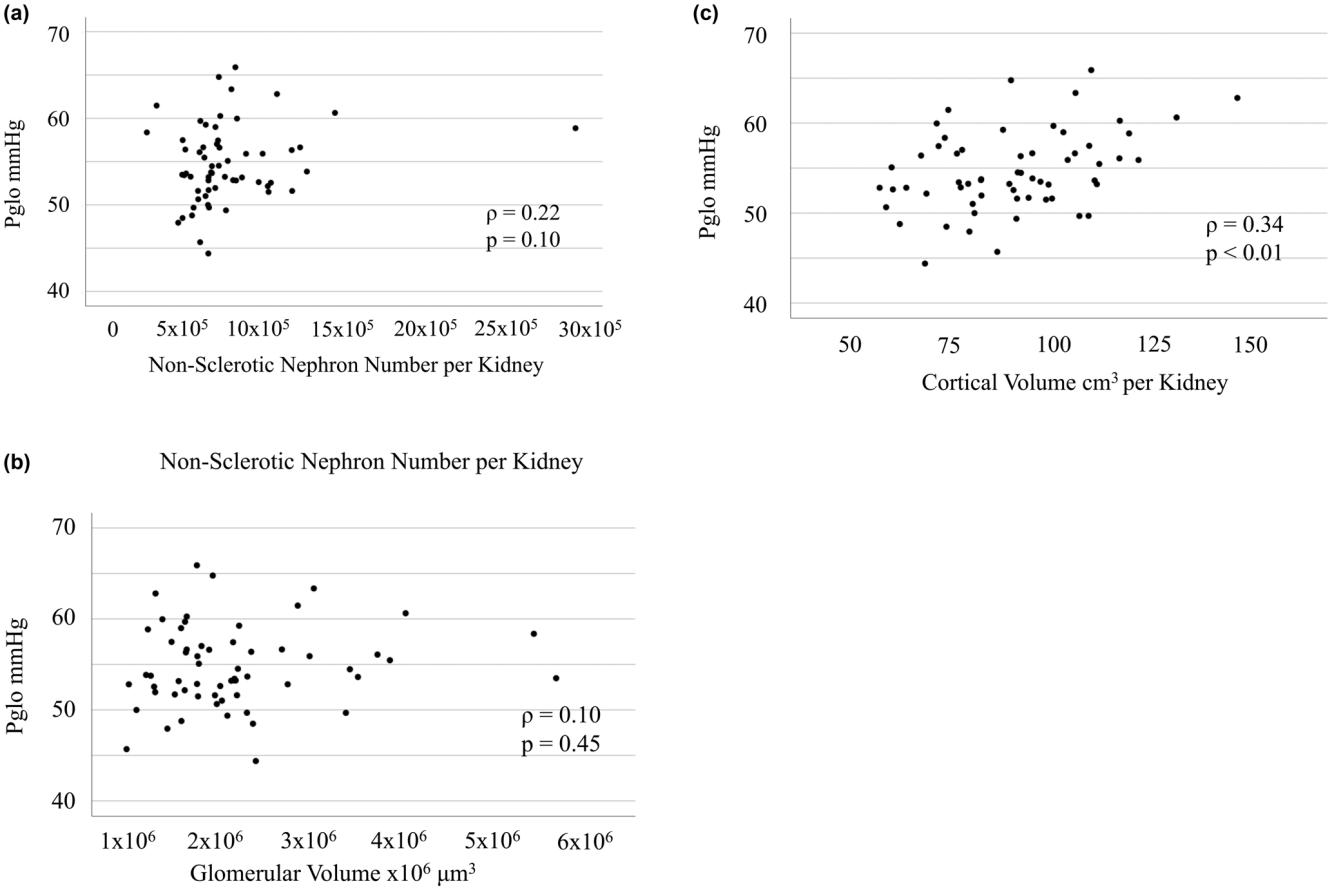
Correlation between Pglo and renal morphological parameters. Association between Pglo and (a) Nglom_NSG_, (b) glomerular volume, and (c) cortical volume. Only cortical volume correlated with Pglo. Pglo, intraglomerular hydrostatic pressure.

Table 2 presents the histopathological characteristics and morphometric data. The median Nglom_TOTAL_ and Nglom_NSG_ in the overall cohort were 699,000 and 659,000, respectively. In the comparison among the three groups based on Pglo levels, the low Pglo group showed lower Nglom_NSG_, higher glomerular density, and lower cortical volume than the other groups, although these differences were not statistically significant. In the correlation analyses, Nglom_NSG_ and glomerular volume showed no correlation with Pglo (*ρ* = 0.22, *p* = 0.10; *ρ* = 0.10, *p* = 0.45, respectively), whereas cortical volume exhibited a significant positive linear correlation (*ρ* = 0.34, *p* < 0.01) (Figure 2a–c).

**TABLE 2:** Histopathological characteristics and morphometric data of all participants according to Pglo tertiles.

	All *n* = 60	Low Pglo (46.6–51.4 mmHg) (*n* = 20)	Intermediate Pglo (51.5–55.8 mmHg) (*n* = 20)	High Pglo (55.8–78.4 mmHg) (*n* = 20)	*p* Value
Nglom_TOTAL_, x10^5^ /kidney	6.99 (6.02–9.52)	7.04 (6.26–9.96)	6.90 (5.76–8.51)	6.99 (5.78–10.49)	0.80
Nglom_NSG_, x10^5^ /kidney	6.59 (5.69–8.08)	6.25 (5.59–9.13)	6.48 (5.76–8.28)	6.90 (5.78–8.07)	0.79
Glomerular volume, x10^6^ μm^3^	1.99 (1.63–2.36)	1.89 (1.40–2.18)	2.19 (1.80–3.26)	1.88 (1.64–2.59)	0.09
Total glomerular volume, cm^3^/kidney	14.3 (11.1–17.0)	12.4 (8.9–16.5)	15.8 (13.6–20.4)	13.5 (11.3–15.6)	0.05
Glomerular density, mm^2^	1.5 (1.1–2.0)	1.7 (1.3–2.2)	1.5 (1.1–2.0)	1.4 (1.1–1.9)	0.24
Cortical volume, cm^3^/kidney	89.1 (74.8–103.5)	79.9 (66.8–91.7)	90.5 (78.2–107.1)	96.0 (73.0–107.9)	0.07
Global sclerosis, %	0 (0–9)	0 (0–15)	0 (0–0)	0 (0–9)	0.06
IF/TA, %	5 (0–5)	5 (5–10)	5 (0–5)	5 (0–5)	0.27

Abbreviations: IF/TA, interstitial fibrosis and/or tubular atrophy; Nglom_NSG_, number of non–sclerosed glomeruli; Nglom_TOTAL_, total nephron number; Pglo, intraglomerular hydrostatic pressure.

### 3.3 Linear regression analyses

To confirm the significant and independent parameters associated with intraglomerular hemodynamics, multivariable linear regression analyses were performed. Pglo, Ra, Re, Nglom_NSG_, glomerular volume, and cortical volume, which did not demonstrate normality, were log‐transformed. Cortical volume showed a positive linear association with Pglo (standardized regression coefficient [*β*] = 0.34, *p* = 0.04) and a negative linear association with Ra (*β* = −0.33, *p* < 0.01). In contrast, Nglom_NSG_ exhibited a positive linear association with Re (*β* = 0.33, *p* = 0.047) (Table 3). Additionally, a multivariable linear regression analysis was conducted using the total glomerular volume per kidney as a covariate instead of a single glomerular volume. In this analysis, cortical volume also demonstrated a positive linear association with Pglo (*β* = 0.34, *p* = 0.04) and a negative linear association with Ra (*β* = −0.32, *p* < 0.01).

**TABLE 3:** Clinicopathological factors associated with Pglo by multivariable linear regression analysis.

	Pglo, mmHg		Ra, dyne·s·cm^−5^		Re, dyne·s·cm^−5^	
	β	*p* Value	β	*p* Value	β	*p* Value
Age, years	−0.12	0.41	0.16	0.09	0.13	0.36
Sex, (male = 1, female = 0)	−0.20	0.16	−0.11	0.24	−0.20	0.17
BMI, kg/m^2^	0.03	0.82	0.02	0.84	0.02	0.90
SBP, mmHg	0.08	0.60	0.73	<0.01	0.10	0.54
Nglom_NSG_, ×10^5^ /kidney	0.12	0.44	0.21	0.06	0.33	0.047
Glomerular volume, ×10^6^ μm^3^	0.17	0.26	0.08	0.40	0.17	0.26
Cortical volume, cm^3^/kidney	0.34	0.04	−0.33	<0.01	−0.15	0.36

*Note*: Pglo, Ra, Re, Nglom_NSG_, glomerular volume, and cortical volume were log‐transformed.

Abbreviations: BMI, body mass index; Nglom_NSG_, number of non‐sclerosed glomeruli; Pglo, intraglomerular hydrostatic pressure; Ra, afferent arteriolar resistance; Re, efferent arteriolar resistance; SBP, systolic blood pressure.

## 4 DISCUSSION

Our findings indicate that Pglo is associated with cortical volume, rather than Nglom, Vglom, or total glomerular volume per kidney, in living kidney donors. This suggests that non‐glomerular morphological factors, particularly tubular volume, play a more significant role in regulating glomerular hemodynamics, potentially surpassing the influence of nephron number alone. An elevated Pglo level has been identified as a key contributor to glomerular hyperfiltration, a process that increases podocyte stress and promotes progressive nephron injury (Yoshida & Shibata, 2024). Although our cohort comprised generally healthy individuals with preserved renal function, the observed variations in Pglo highlighted the presence of subclinical differences in Pglo.

A key finding of this study is that cortical volume, rather than Nglom or Vglom, is significantly correlated with both Pglo and Ra. Since the non‐glomerular tissue within the renal cortex consists predominantly of tubules, this suggests that tubular volume within the cortex may influence intraglomerular hemodynamics, potentially through modulation of afferent arteriolar tone. The negative correlation between cortical volume and Ra implies that tubular hypertrophy may reduce RA, thereby promoting glomerular hyperfiltration. These results, in part, could be explained by the tubular hypothesis, which states that the increased workload of the proximal tubules, including glucose reabsorption, leads to an enlargement of the proximal tubular volume, which is reflected in the kidney enlargement observed during the early stages of diabetic kidney disease (Vallon & Thomson, 2020). According to this hypothesis, enhanced proximal tubular reabsorption induces both tubular hypertrophy and hyperfiltration by reducing Ra through the attenuation of tubulo‐glomerular feedback. In addition, tubular hypertrophy and enhanced reabsorption reduce the hydrostatic pressure in Bowman's space by lowering the flow rate through distal nephron segments where flow resistance is high. This reduction in the hydrostatic pressure in Bowman's space results in the glomerular hyperfiltration by increasing the effective glomerular filtration pressure (Hallow et al., 2017; Vallon, 2011).

Importantly, our findings support a model in which early tubular compensation may initially maintain glomerular filtration, but when persistent, may also predispose to progressive nephron injury due to sustained hyperfiltration stress. Notably, although the participants were nondiabetic kidney donors, previous studies have reported a positive correlation between Pglo and insulin resistance, even in healthy kidney donors (Tsuda et al., 2018). This raises the possibility that individuals with increased cortical volume may exhibit subtle metabolic alterations even in the absence of overt hyperglycemia, which warrants further investigation.

A particularly intriguing finding is that Nglom_NSG_ is positively correlated with Re. This implies that a lower number of non‐sclerotic glomeruli was associated with a reduction in Re and, consequently, a decline in glomerular filtration. This contrasts with the classical Brenner hypothesis, which proposed that a reduction in glomerular number leads to compensatory hyperfiltration in the remaining nephrons (Brenner et al., 1988). Recently, it was proposed that with a gradual decline in the number of glomeruli, the total GFR decreases, whereas the single‐nephron GFR remains constant unless the number of glomeruli declines below a certain threshold (Fattah et al., 2019). Additional studies by Denic et al. suggested that although glomeruli size appeared not to change, tubular size increased with age‐related nephron loss in humans (Denic et al., 2016; Denic, Lieske, et al., 2017). These findings suggest tubular compensation may serve as an early adaptive mechanism to mitigate the hemodynamic consequences of nephron loss. Our findings support a revised model in which moderate nephron loss does not necessarily lead to immediate hyperfiltration in the remaining glomeruli. Instead, tubular hypertrophy may mitigate the effects of nephron loss and maintain renal function. However, prolonged tubular compensation may eventually result in hyperfiltration‐mediated injury, as evidenced by the association between cortical volume and Pglo. These findings provide a framework for reconsidering the interplay among nephron loss, tubular adaptation, and intraglomerular hemodynamics.

Although eGFR is widely applied in clinical practice to assess renal function and define CKD stages, it does not provide insight into the functional burden placed on the individual nephrons. Our results indicate that kidney donors with a normal eGFR may exhibit significantly elevated Pglo, suggesting the presence of mild hyperfiltration at the single‐nephron level. Furthermore, our findings suggest that the cortical volume may serve as an indirect marker of glomerular hyperfiltration. If validated in larger studies, imaging‐based cortical volume assessments could provide an early indicator of nephron‐level hyperfiltration risk even in individuals with seemingly normal eGFR. This would have important implications for identifying patients at risk of CKD progression before an overt GFR decline is observed.

Clinically, our results highlight the potential value of assessing Pglo even in individuals with preserved renal function. Measuring cortical volume using noninvasive and accessible ultrasound techniques may contribute to the early identification of hyperfiltration and guide interventions aimed at reducing Pglo, such as pharmacological approaches (e.g., renin‐angiotensin‐aldosterone system inhibitors and sodium‐glucose cotransporter‐2 inhibitors).

This study had some limitations that must be acknowledged. First, Pglo was estimated using Gomez's equation, which relies on specific assumptions and population‐averaged values. Although this method provides a noninvasive approach to evaluating Pglo, direct in vivo measurement remains challenging in human studies. Second, we substituted BSA‐unadjusted eGFR for GFR in the calculation of the Gomez formula, which is less accurate than GFR measured by inulin clearance. However, the median Pglo in our study differed by only 1.1% from the mean Pglo calculated using inulin clearance–based GFR in healthy Japanese living kidney donors (Tsuda et al., 2018). Therefore, eGFR may be considered a reasonable surrogate for directly measured inulin clearance, which is a complex and invasive procedure for patients. Third, our sample size was relatively small and included only Japanese kidney donors with normal renal function, which limits the generalizability of our findings. Further studies in larger, multiracial cohorts are required to confirm these associations in diverse populations. Fourth, this was a cross‐sectional observational study; therefore, no causal relationship could be established between intraglomerular hemodynamic parameters and renal morphological factors. Prospective studies and basic experimental research are required to investigate the relationships between parameters and further explore the underlying physiological mechanisms.

## 5 CONCLUSION

Overall, our findings suggest that Pglo is more dependent on cortical volume than on the absolute number of glomeruli in healthy kidney donors. This supports a model in which tubular hypertrophy modulates Ra, thereby contributing to glomerular hyperfiltration. Clinically, these results underscore the need to assess intraglomerular hemodynamics and cortical volume as potential markers for early CKD risk beyond traditional eGFR measurements. Future research should aim to validate these findings in larger, more diverse cohorts and explore potential interventions that may mitigate the adverse effects of early glomerular hyperfiltration.

## AUTHOR CONTRIBUTIONS

H.N. and G.K. were involved in conceptualization, methodology, formal analysis, investigation, data curation, writing—original draft preparation. T.Y. was involved in resources and supervision. H.N., G.K., R.O., K.H., T.S., Y.O., K.K., N.T., and T.Y. were involved in writing—review and editing. H.N. was involved in visualization. G.K. was involved in project administration. All authors contributed to the data interpretation and approved the final version of the manuscript.

## FUNDING INFORMATION

This work was supported by a Japan Kidney Foundation Research Grant and the Japan Society for the Promotion of Science (JSPS) through the Grants‐in‐Aid for Scientific Research (KAKENHI) grant numbers JP18K15987 and JP21K16197 (to Go Kanzaki).

## CONFLICT OF INTEREST STATEMENT

The authors declare that they have no conflicts of interest.

## ETHICS STATEMENT

This study was approved by the ethics review board of Jikei University School of Medicine (approval no. 30–060, 9081).

## INFORMED CONSENT

Information on the research plan and possibility of publication was proposed, and an opportunity to opt out was provided; therefore, individual informed consent was not required.

## Data Availability

The datasets used and analyzed during this study are available from the corresponding author upon reasonable request.
